# Supplementary material for: Acceptance and compliance with micronutrient powder (MNP) among children aged 6–23 months in northern Nigeria
Source: PLOS Glob Public Health. 2022 Oct 17;2(10):e0000961. doi: 10.1371/journal.pgph.0000961 (PMC10022258; doi:10.1371/journal.pgph.0000961)
Supplement: S1 Dataset — (ZIP) [file pgph.0000961.s008.zip › S8_Dataset/Spot Checks.zip!/Spot Checks/13072015_A_GU_AD_2_SC.pdf]

SPOT CHECK RECORDING FORM

State: Adamawa _ LGA:_Guyuk__________ Community: lokoro_

Name of caregiver (female):……..Sarauniya Augustine…….

Name of caregiver (male): ……………….……………….._

Sex of index child: male___ Age of index child (in months): 18 month________

Spot Check Information

1. **Date of MNP distribution to household** (circle option below based on LGA name above)**:**
   - **Kebbi:** BirninKebbi&Jega LGA: **June 16**
   - **Kebbi:** Danko-wasagu: **June 17**
   - **Adamawa:**Song, Ganye: **June 18**
   - **Adamawa:**Guyuk **June 19**
2. **Date of Spot Check Observation: ___13/07/2015_____**
3. **Number of sachets remaining**(Ask the caregiver to show you and count the specific number of sachets remaining)**:**

   35

4. **Number of sachets that should remainusing one MNP sachet per day** (Analyst only)**:**

   37

5. **Number of sachets remaining / number of sachets should be remaining**(Analyst only)**:**

   _ 35__ / ___37_____ = __94.6_______ %

Note

The mother said she was given MNP on the 17th of June

Probes

Question 1. You have **XX** MNP sachets remaining. Explain how easy or difficult it was to use the MNP product on a daily basis for your child 6 – 23 months? (Circle one answer)

Very Difficult **Difficult** **Easy** **Very Easy**

Question 2. Describe any specific challenges that you faced that made the MNP difficult to use on a daily basis?

I have to use my teeth to tear off the sachet. Preparing kunu to administer the MNP with on the daily basis has not been an easy take. He has diarrhea when I started given him the MNP

Question 3
He always finishes the kunu mixed with MNP with ease
He is healthier and full of energy

Question 4. What questions do you have about the MNP based on your experience using it?

Dose this MNP has side effect .
Can I take a break from using the MNP because of the diarrhea

Question 5. What specific recommendations do you have that we can do as a program to make the MNP easier to use on a daily basis?

I had doubt about the MNP and I behaved some caregivers feel the same. There is need for adequate education on MNP in this community. Because my opinion is those who brought this MNP must have thought our children need it and has our best interest at that is why we were chosen to get it.
